# Supplementary figures and images for: Progressive Mitochondrial SOD1G93A Accumulation Causes Severe Structural, Metabolic and Functional Aberrations through OPA1 Down-Regulation in a Mouse Model of Amyotrophic Lateral Sclerosis
Source: Int J Mol Sci. 2021 Jul 30;22(15):8194. doi: 10.3390/ijms22158194 (PMC8347639; doi:10.3390/ijms22158194)

**A**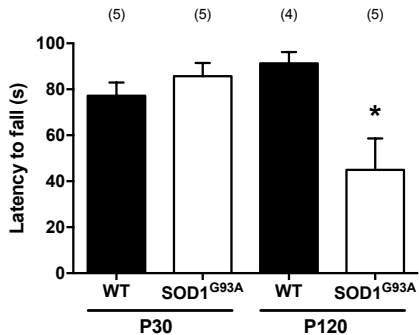**B**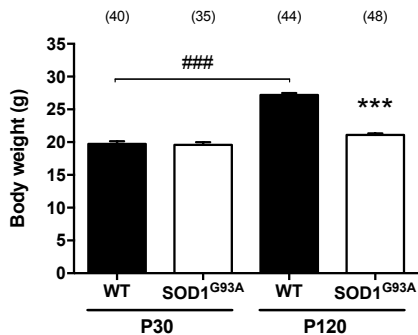

**Supplementary figure S1**

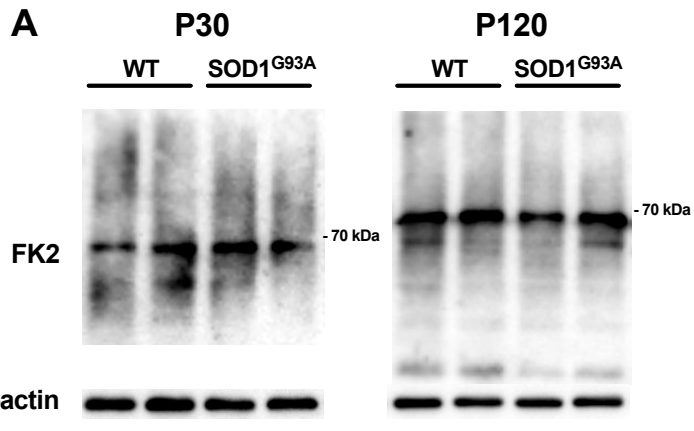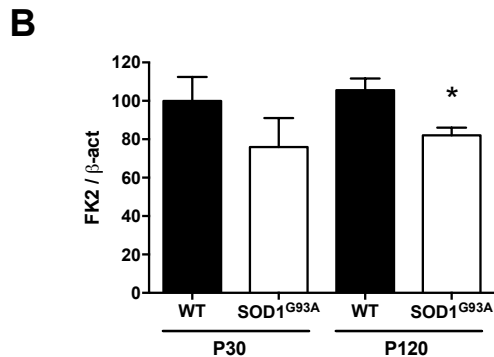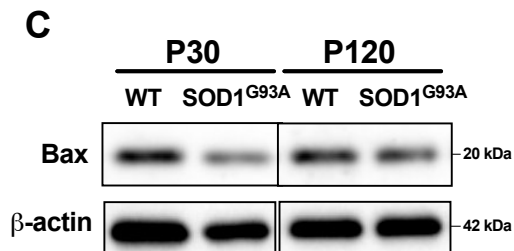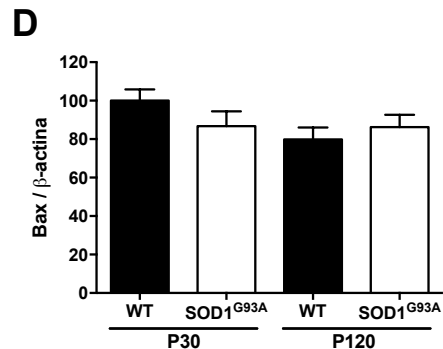

**Supplementary figure S2**

Supplement: Supplementary file 1 [file ijms-22-08194-s001.zip › ijms-1286071-supplementary.pdf]
